# A female with fever and shortness of breath

**DOI:** 10.4103/1817-1737.43086

**Published:** 2008

**Authors:** Saurabh K. Singh, Zuber Ahmad, Rakesh Bhargava, Deepak K. Pandey

**Affiliations:** *Department of TB and Respiratory Diseases, JN Medical College, AMU Aligarh - 200 002, UP, India*

A 26-year-old female presented with the complaints of fever and cough of 1 month's duration. For the last 3 days she had also had increased shortness of breath. On examination, her temperature was 101.2°F, pulse rate was 96/min, and respiratory rate was 20/min. Her nutritional status was poor and she was anemic. On examination of her chest, crepitus was felt at the root of the neck and over the right anterior chest wall. On auscultation, a crunching sound was heard over the precordium, which was synchronous with the heart sounds. Fundus examination showed miliary tubercles. Blood investigations were unremarkable, except for hemoglobin of 8 g/dL. A chest X-ray, PA view, was obtained and is shown in Figure 1.

**Figure 1:**
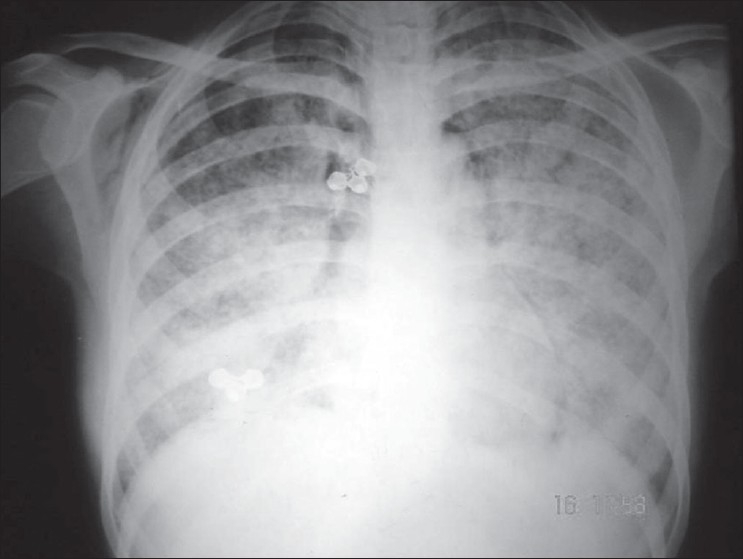
Chest X-ray

## Questions

1. What is the radiological finding?
2. How common are miliary tubercles in the fundus in such cases?
3. Explain the mechanism of this complication.

## Answers

The chest radiograph shows the presence of coarse miliary tubercles in both lung fields. It also shows subcutaneous emphysema on the right side, along with a linear opacity paralleling the left heart border; however, there is no evidence of pneumothorax. A diagnosis of miliary tuberculosis, with pneumomediastinum and subcutaneous emphysema, was made. Antitubercular drugs (rifampicin, INH, pyrazinamide, and ethambutol) given in standard doses for 6–9 months is the treatment of choice. Oxygen and analgesics can be given as supportive therapy. Miliary tubercles on fundus examination are found in only 4.5% of such cases.[1] The mechanism of development of pneumomediastinum in case of miliary tuberculosis is discussed in the following section.

## Discussion

Pneumomediastinum (mediastinal emphysema) is defined as the presence of air in the mediastinum. Gas at this site can originate from any of the following[2]: (i) mediastinal airways or the esophagus, (ii) neck along the fascial planes (iii) abdominal cavity, and (iv) interstitial tissue of the lung.

The lung is the commonest source of the air in the mediastinal space. This occurs due to a sudden increase in alveolar pressure causing it to rupture. Rupture results in the escape of air into the perivascular or peribronchial interstitium from where the air reaches the hilum and the mediastinal space. This air can now track upwards along the fascial planes to reach the neck, or along the sheath of the aorta to reach the abdomen. The air may rupture mediastinal pleura and cause pneumothorax.

Pneumomediastinum is clinically characterized by chest pain and dyspnea. Physical examination usually reveals the subcutaneous emphysema over the neck and anterior chest wall. A crunching sound that is synchronous with heart sounds can be heard on auscultation over the chest and is known as Hamman's sign. In one study the prevalence of this sign in pneumomediastinum was found to be 10%.[3] Radiologically, pneumomediastinum shows as radiolucent streak of air outlining the mediastinal structures due to displacement of mediastinal pleura.

Uncontrolled hematogenous dissemination of *Mycobacterium tuberculosis* may lead to miliary tuberculosis. The mechanism of occurrence of pneumomediastinum and subcutaneous emphysema in our patient was probably due to the excessive coughing associated with miliary tuberculosis; alveolar rupture due to the sudden increase in intra-alveolar pressure with concomitant airway narrowing would have forced air into the interstitial tissues of the lung and, thence, into the vascular adventitia of the hilum.

There are very few case reports that have described pneumomediastinum with subcutaneous emphysema, but without pneumothorax, occurring as a complication of miliary tuberculosis[4] However, there are case reports describing the occurrence of pneumothorax in miliary tuberculosis, which is itself is a rare entity.[5]
